# Translation Inhibitors Activate Autophagy Master Regulators TFEB and TFE3

**DOI:** 10.3390/ijms222112083

**Published:** 2021-11-08

**Authors:** Thao Thi Dang, Sung Hoon Back

**Affiliations:** School of Biological Sciences, University of Ulsan, Ulsan 44610, Korea; dangthaosphn@gmail.com

**Keywords:** autophagy-lysosome pathway, TFEB, TFEB nuclear translocation, mTOR, calcineurin, ribosome, eIF4A helicase, translation inhibitor

## Abstract

The autophagy-lysosome pathway is a major protein degradation pathway stimulated by multiple cellular stresses, including nutrient or growth factor deprivation, hypoxia, misfolded proteins, damaged organelles, and intracellular pathogens. Recent studies have revealed that transcription factor EB (TFEB) and transcription factor E3 (TFE3) play a pivotal role in the biogenesis and functions of autophagosome and lysosome. Here we report that three translation inhibitors (cycloheximide, lactimidomycin, and rocaglamide A) can facilitate the nuclear translocation of TFEB/TFE3 via dephosphorylation and 14-3-3 dissociation. In addition, the inhibitor-mediated TFEB/TFE3 nuclear translocation significantly increases the transcriptional expression of their downstream genes involved in the biogenesis and function of autophagosome and lysosome. Furthermore, we demonstrated that translation inhibition increased autophagosome biogenesis but impaired the degradative autolysosome formation because of lysosomal dysfunction. These results highlight the previously unrecognized function of the translation inhibitors as activators of TFEB/TFE3, suggesting a novel biological role of translation inhibition in autophagy regulation.

## 1. Introduction

Macroautophagy, hereafter referred to as autophagy, is a normal degradative pathway that exists in all eukaryotic cells [1,2]. Autophagy involves sequestration of cytoplasmic contents, including organelles, by double membranes, to form a unique nascent autophagic vacuole (hereafter autophagosome) and their delivery to lysosomes for digestion [3,4,5]. A number of autophagy-related proteins are implicated in the formation of the autophagosome, such as microtubule-associated proteins 1A/1B light chain 3B (LC3B). LC3B produced as a precursor (pro-LC3B) is cleaved by the ATG4 protease into a cytosolic form referred to as LC3B(I). LC3B(I) is subsequently conjugated with phosphatidylethanolamine (PE) to LC3B(II) via a ubiquitination-like enzymatic reaction, followed by insertion into both inner and outer membranes of the growing vesicular sac (also called phagophore). Consequently, increased LC3B(II) is routinely used as a marker of autophagy activation. In addition, LC3B is widely used as a microscopic marker of phagophores and autophagosomes [6,7]. 

The progression and resolution of autophagy critically depends on the lysosomal function, as lysosomes play a role in the breakdown and recycling of cellular compartments. Lysosome is a single membrane-bound compartment that is filled with more than 60 resident acid hydrolases: proteases, phosphatases, lipases, nucleases, and glycosidases [8,9]. Most of these enzymes are functionally optimized at a low pH, which is maintained by the vacuolar H^+^-ATPase (V-ATPase), an ATP-driven proton pump located on the lysosomal transmembrane [10,11]. Lysosome mainly contains various acidic proteases, including cathepsins representing a major class of lysosomal proteases, which contribute to the degradation of proteins or organelles. The cathepsin family consists of three different protease families including aspartic proteases (cathepsin D and E), serine proteases (cathepsins A and G), and cysteine cathepsins (cathepsins B, C, F, H, K, L, O, S, V, X, and W) [12,13]. Cathepsins are synthesized as inactive pro-cathepsins in the endoplasmic reticulum (ER) and transported into the endosome/lysosome compartment. Inside lysosomes, the cleavage of propeptide converts pro-cathepsins to mature active cathepsins [13]. Most lysosomal cathepsins are functionally optimized at a low pH, as cathepsins are stable and active at an acidic pH. The most abundant lysosomal membrane proteins include the lysosome-associated membrane protein (LAMP)1 and LAMP2, which together constitute ~50% of lysosomal membrane proteins [14,15]. Therefore, those proteins are used as markers of the lysosome level and integrity. 

The induction and formation of autophagosome during autophagy are followed by late stages such as autophagosome-lysosome fusion and cargo degradation for completion of autophagy [4,5,11]. The fusion of nascent autophagosome with late endosome or lysosome generates autolysosome, a process also known as autophagosome maturation, which is mediated by ATG8 family members, membrane tethering complexes, Rab GTPases, soluble N-ethylmaleimide-sensitive factor attachment protein receptors (SNAREs), and a voltage-gated calcium channel [4,5,16,17]. The lysosomal V-ATPase is responsible for lysosome acidification and is indispensable for lysosomal acid enzyme activation and cargo degradation. However, V-ATPase-deficient lysosomes can fuse with autophagosomes and endosomes [18]. In addition, Niemann–Pick type C disease (NPC) cells and CtsB/L inhibition or genetic depletion can impair autolysosome clearance but preserve intact autophagosome-lysosome fusion [19,20]. Thus, the studies suggest that intact lysosomal acidification and protease activity are not required for autolysosome formation although lysosomal impairment can lead to an accumulation of cargo in inactive autolysosomes.

Autophagy was considered as a pathway exclusively regulated by cellular processes in enucleated cells forming autophagosomes [21]. However, increasing evidence indicates that autophagy is regulated at the transcription level by several transcriptional factors including [transcription factor EB (TFEB), transcription factor E3 (TFE3), E2 transcription factor1 (E2F1), and Forkhead box O (FOXO)] [22,23,24]. TFEB is a member of the microphthalmia-associated transcription factor (MiTF) that also includes MITF, TFE3, and TFEC proteins [25]. It is believed that TFEB and TFE3 are master regulators of the autophagy-lysosome pathway (ALP) controlling the expression of genes required for autophagosome formation, lysosome biogenesis, and lysosome function [26,27,28]. Activities of TFEB and TFE3 are regulated by post-translational modification, especially phosphorylation [29,30,31,32]. To date, several kinases that phosphorylate TFEB and TFE3 have been identified. Among them, mTOR, as part of the protein complex Torin-2-mediated mechanistic target of rapamycin complex 1 (mTORC1), represents the main kinase responsible for TFEB and TFE3 phosphorylation [33,34,35,36]. Under nutrient-rich conditions, the lysosome-localized mTOR phosphorylates TFEB (at Ser142 and Ser211) and TFE3 (at Ser321). The phosphorylated TFEB and TFE3 subsequently interact with the cytosolic chaperone 14-3-3 proteins, which results in sequestration of these transcription factors as an inactive form in the cytosol [33,35,36,37]. Under nutrient deprivation and metabolic stress, mTOR activity is inhibited and/or Ca^2+^-calmodulin-dependent protein phosphatase calcineurin is activated, without further phosphorylation of TFEB and TFE3, resulting in the prevention of binding to 14-3-3 and rapid accumulation of TFEB and TFE3 in the nucleus [30,38,39,40]. However, recent studies have shown that mTOR-independent phosphorylation (S138 and S134) and calcineurin-independent dephosphorylation also play a role in the modulation of TFEB localization, indicating that other kinases and phosphatases can regulate TFEB activity [41,42,43]. Thus, the mechanisms governing TFEB/TFE3 localization in response to multiple signals are still not fully understood. Subsequently, nuclear TFEB promotes the transcription of genes required for autophagosome formation, lysosome biogenesis and lysosome function by direct binding to promoters of the coordinated lysosomal expression and regulation (CLEAR) element [26,27,28].

Protein biosynthesis is one of the major metabolic processes, which are crucial for maintaining cellular functions including autophagy. Therefore, eukaryotic translation is an attractive target to destroy fast-growing tumor cells and impair and/or delay the spread of fast-replicating viral pathogens [44,45,46,47,48]. In addition, growing evidence indicates that autophagy modulation is also important for anti-cancer [49,50] and anti-viral therapies [51,52]. The mTORC1 not only regulates autophagy via TFEB/TFE3 phosphorylation [33,34,35,36], but also stimulates mRNA translation via phosphorylation of several translational regulatory proteins such as eukaryotic translation initiation factor 4E (eIF4E)-binding proteins 1 and 2 (4E-BP), ribosomal protein S6 kinases 1 and 2 (S6K1/2), and RNA-binding protein La-related protein 1 (LARP1) [53]. It is known that mTORC1 inhibitors such as Torin1 and 2 can inhibit mRNA translation [47,54] but induce autophagy [29,30,33,34,35,36]. Therefore, we investigated whether other translation inhibitors can also affect TFEB/TFE3 phosphorylation, localization, and further autophagic pathways. In this report, we analyzed three different translation inhibitors with different mechanisms of action. First, we used cycloheximide (CHX), a well-known translation inhibitor that can bind to the E-site of the 60S ribosome together with deacylated tRNA, followed by ribosome arrest on the next codon in the mRNAs anywhere [47,55,56]. The next inhibitor was another 60S tRNA E-site inhibitor lactimidomycin (LTM), which can preferentially arrest ribosome at the first peptide bond [47,55,56]. The last one was a natural product rocaglamide A (RocA) isolated from plants belonging to genus *Aglaia*. It is an inhibitor of eukaryotic initiation factor 4A (eIF4A), an ATP-dependent DEAD-box RNA helicase. It preferentially represses translation by clamping eIF4A onto purine-rich regions within mRNA 5′ leaders, followed by inhibition of 43S pre-initiation complexes (PICs), leading to the premature translation from uORF and the inhibition of downstream ORF translation [47,57,58]. Further, a recent study suggested that rocaglates including RocA can interfere with the heterotrimeric (eIF4A, 4E, and 4G) eIF4F complex release from the cap (7-methylguanosine) structure, resulting in direct inhibition of translation of the target mRNA and a bystander effect that leads to trans-inhibition of translation on otherwise normally unresponsive mRNAs [59]. Among these inhibitors, it has been reported that CHX pretreatment under starvation conditions cannot inhibit the formation of autophagosomes, but can prevent their conversion to degradative autolysosomes [60], although conflicting reports of its effect on autophagy exist [61,62]. However, whether these translation inhibitors can modulate the localization of autophagy master regulators TFEB/TFE3 and whether translation inhibition can affect autophagic pathways require further investigation.

In the present study, we analyzed the impact of translation inhibition using three chemicals (CHX, LTM, RocA) on TFEB localization and autophagy pathways. Such chemical-mediated translation inhibition promoted TFEB dephosphorylation and nuclear translocation. TFEB nuclear localization was modulated by a phosphatase calcineurin but not a kinase mTORC1. In addition, LTM and RocA significantly increased the expression of a number of genes downstream of TFEB required for autophagosome formation, lysosome biogenesis, and lysosome function. Further, we demonstrated that treatment with LTM and RocA facilitated autophagosome biogenesis but prevented degradative autolysosome formation.

## 2. Results

### 2.1. Glutarimide-Containing Ribosome Inhibitors and eIF4A Helicase Inhibitor-Mediated Translation Inhibition Induce Transcription Factor EB (TFEB) and Tanscroption Factor E3 (TFE3) Nuclear Translocation

To analyze changes in TFEB cellular localization under diverse experimental conditions, we established a mouse embryonic fibroblast (MEF) cell line expressing a human TFEB-fused enhanced green fluorescent protein (EGFP) at the C-terminal and a control MEF cell line expressing EGFP only (Supplemental Figure S1A,B). As it is known that most endogenous TFEB is observed in the cytoplasm before activation [23,24]. Our results showed that TFEB-EGFP was mainly localized in the cytoplasm, whereas EGFP alone was distributed in the cell under basal conditions (Supplemental Figure S1A). Next, we determined whether TFEB-EGFP nuclear translocation can be induced by starvation or mechanistic target of rapamycin complex 1 (mTORC1) inhibition in the established MEF-TFEB-EGFP cell line. As reported previously [23,24,28,36], most cells carried nuclear translocated TFEB-EGFP in both EBSS-mediated starvation (Supplemental Figure S1C,D) and Torin-2-mediated mTORC1 inhibition (Supplemental Figure S1F,G) conditions. In addition, this change in subcellular localization under starvation or mTORC1 inhibition is paralleled by TFEB dephosphorylation [33,34,35]. Therefore, similar to previous reports [33,34,35], inhibition of mTORC1 by treatment with starvation or Torin2 altered the electrophoretic mobility of TFEB-EGFP including endogenous TFEB, which appeared as fast-migrating forms (Supplemental Figure S1E,H). Based on these results, the established MEF-TFEB-EGFP cell line can be used as a reporter cell line to monitor TFEB subcellular localization.

**Figure 1:**
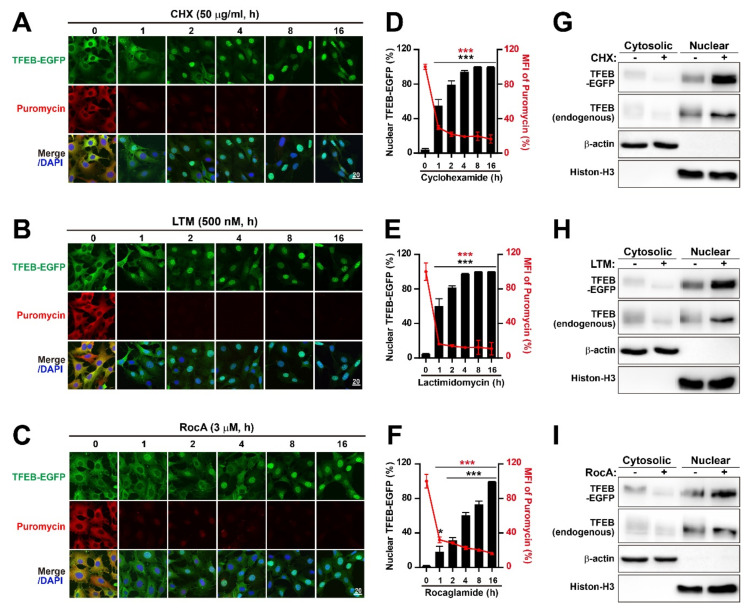
Translation inhibition by cycloheximide (CHX), lactimidomycin (LTM) and rocaglamide A (RocA) induces nuclear translocation of TFEB. (**A**–**C**) Representative images of confocal analysis. TFEB-EGFP expressing MEF (MEF-TFEB-EGFP) cells were treated with cycloheximide (CHX, 50 μg/mL) (**A**), lactimidomycin (LTM, 500 nM) (**B**) rocaglamide A (RocA, 3 μM) for the indicated times. Before harvesting, cells were additionally incubated with puromycin (10 μg/mL for 10 min) to label actively translating peptides. Cells were stained with anti-puromycin antibody (red) against puromycin-labeled proteins and DAPI (blue) for nucleus DNA. Cellular localization of TFEB-EGFP was indicated by green fluorescence signals in the cells. Scale bar, 20 μm. (**D**–**F**) Quantification of the percentage of MEF-TFEB-EGFP cells with nuclear TFEB-EGFP (left *y* axis) and the mean fluorescent intensity (MFI) of puromycin (right *y* axis) following CHX, LTM or RocA treatment as indicated in (**A**–**C**). Data are expressed as mean ± SEM of at least 150 cells from 6 random fields in each group. *** *p* < 0.001; 0 h vs. other times. (**G**–**I**) Immunoblot analysis of subcellular distribution of TFEB-EGFP and endogenous TFEB in MEF-TFEB-EGFP cells treated with or without CHX (50 μg/mL) (**G**), LTM (500 nM) (**H**) or RocA (3 μM) (**I**) for 8 h was performed, using antibodies against GFP and TFEB. β-actin and Histon-H3 were used as loading controls of cytoplasmic and nuclear fractions, respectively.

Next, we determined whether the inhibition of protein synthesis induced TFEB nuclear translocation in the MEF cell line, by treating MEF-TFEB-EGFP cell lines with glutarimide-containing natural products cycloheximide (CHX) and lactimidomycin (LTM) in a dose (Supplemental Figure S2A–D) and time (Figure 1A,B,D,E) dependent manner, respectively. To assess the impact of the inhibitors on translation, we performed puromycin labeling, followed by immunofluorescence using an antibody against puromycin in cells treated with or without inhibitors (Figure 1A,D and Supplemental Figure S2A,C). Fluorescence intensities of puromycylated nascent peptides were strong in cells treated without CHX. The intensity was significantly reduced by CHX in a dose-dependent manner. It declined eventually to ~20% by treatment with 50 μg/ml CHX, suggesting that the working concentration range of CHX was sufficient to observe its inhibitory effect on cellular translation (Supplemental Figure S2A,B). Under similar treatment conditions of CHX, a green fluorescence signal of EGFP was observed to determine the subcellular localization of TFEB-EGFP. The CHX treatment at the lowest concentration (2 μg/mL) for 4 h even induced nuclear translocation of TFEB-EGFP in almost 80% of cells (Supplemental Figure S2A,B) although a minor fluorescence signal of TFEB-EGFP was still observed in the cytoplasm. However, the increase in CHX concentration to 50 μg/mL resulted in the nuclear translocation of almost all TFEB-EGFP proteins (Supplemental Figure S2A,B). Next, in a time-course experiment, the nuclear translocation of TFEB-EGFP in approximately 55% of MEF-TFEB-EGFP cells occurred at 1 h after treatment with CHX 50 μg/mL (Figure 1A). The percentage of MEF-TFEB-EGFP cells carrying nuclear TFEB-EGFP reached nearly 100% at 8 h after treatment (Figure 1A,D). To validate our results, we performed a subcellular fractionation analysis using MEF-TFEB-EGFP cells. As expected, we found that the amount of TFEB-EGFP in the nuclear fraction increased upon treatment with CHX (Figure 1G). Furthermore, we observed the augmented nuclear accumulation of endogenous TFEB in response to the CHX treatment (Figure 1G). CHX can bind to the E-site of the 60S ribosome together with deacylated tRNA, resulting in the freezing of all translating ribosomes in the mRNAs [47,56,63]. Therefore, we tested another translocation inhibitor lactimidomycin (LTM). It is known to preferentially act on initiating ribosomes, but not on elongating ribosomes. Thus, its addition to cells can lead to polysome disassembly [47,56,63]. Different doses (100, 250, 500, and 1000 nM for 4 h) of LTM clearly showed its translational inhibitory effects (Supplemental Figure S2C,D). In addition, LTM treatment induced the nuclear translocation of TFEB-EGFP in more than 90% of the cells under all tested concentrations (Supplemental Figure S2C,D). Next, similar to the time-dependent experiment of CHX, the maximal nuclear translocation of TFEB-EGFP (around 100%) occurred at 8 h after MEF-TFEB-EGFP cells were treated with LTM 500 nM (Figure 1B,E). Finally, the subcellular fractionation analysis confirmed the augmented nuclear accumulation of TFEB-EGFP and endogenous TFEB following the LTM treatment (Figure 1H).

Although CHX and LTM are specific inhibitors of protein synthesis blocking the ribosome at the translocation stage, we cannot exclude the possibility that TFEB and TFE3 nuclear translocation might be induced as an unknown side effect. Therefore, a different type of translation inhibitor RocA with distinct mechanism of action was selected to reproduce our observation. RocA, an inhibitor of eukaryotic initiation factor 4A (eIF4A), preferentially represses translation by clamping eIF4A onto purine-rich regions within mRNA 5′ leaders. It then interferes with the scanning of 43S pre-initiation complexes (PICs) [57,58]. Furthermore, a recent report suggested that RocA can interfere with the release eIF4F complex from the cap structure to inhibit the translation of the target mRNA. It exerts a bystander effect on translation initiation by sequestering eIF4F, leading to trans-inhibition of translation on otherwise normally unresponsive mRNAs [59]. Therefore, RocA also acts as a general translation inhibitor similar to CHX and LTM.

First, in dose-dependent experiments (0.5, 1, and 3 μM for 4 h) RocA clearly showed its translational inhibitory effect (Supplemental Figure S2E,F). As expected, RocA treatment gradually increased the nuclear translocation of TFEB-EGFP in a dose-dependent manner. RocA treatment (3 μM for 4 h) induced TFEB-EGFP nuclear translocation of ~77% of cells (Supplemental Figure S2E,F). Next, a time course experiment showed that RocA treatment (3 μM for 16 h) induced nuclear translocation of TFEB-EGFP in almost 100% of cells (Figure 1C,F), suggesting that RocA was also a strong inducer of TFEB nuclear translocation although additional time was needed to attain the maximal level of TFEB nuclear translocation compared with CHX or LTM. Finally, a subcellular fractionation analysis confirmed augmented nuclear accumulation of TFEB-EGFP and endogenous TFEB following RocA treatment (Figure 1I). These results indicate that translation inhibition may induce nuclear translocation of TFEB, a master regulator of lysosome biogenesis and autophagy.

To exclude the nuclear translocation of TFEB in response to three translation inhibitors as a cell type-specific event, we assessed endogenous TFEB nuclear translocation in both mouse immortalized hepatocytes and human HeLa cells [64]. In agreement with results of the MEF-TFEB-EGFP cell line, both immortalized hepatocytes and human HeLa cells showed almost 100% of nuclear translocation upon treatment with CHX, LTM or RocA for 8 h, whereas less than 30% of cells showed accumulation of TFEB in the nucleus under normal conditions (Supplemental Figure S3A,B). TFEB belongs to the MiT family of helix-loop-helix leucine zipper transcription factors, together with TFE3, MITF and TFEC [23,24]. Therefore, the regulatory mechanism of TFEB is shared by TFE3 [23,24,32] and TFEB and TFE3 play a cooperative role as needed [65,66]. Therefore, we asked whether TFE3 can also be translocated to the nucleus in both immortalized hepatocytes and human HeLa cells treated with a translation inhibitor CHX, LTM, or RocA. As expected, similar to TFEB, TFE3 was also efficiently translocated into the nucleus in both immortalized hepatocytes and human HeLa cells treated with CHX, LTM or RocA for 8 h (Supplemental Figure S3C,D). Together, our results suggest that translation inhibition might induce the nuclear translocation of both TFEB and TFE3 via a common regulatory mechanism conserved in different cell types and species.

### 2.2. Translation Inhibitors Induce TFEB Dephosphorylation and 14-3-3 Dissociation

**Figure 2:**
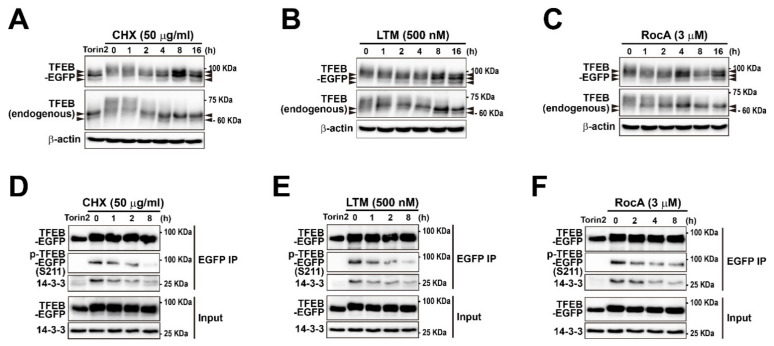
Translation inhibition promotes TFEB dephosphorylation and 14-3-3 dissociation. (**A**–**C**) MEF-TFEB-EGFP cells were treated with CHX (50 μg/mL) (**A**), LTM (500 nM) (**B**), or RocA (3 μM) (**C**) for indicated times and Torin2 (mTOR inhibitor, 100 nM) for 3 h as a positive control. Total cellular lysates were separated on 6% SDS-PAGE, followed by immunoblotting with antibodies against GFP, TFEB (used to detect TFEB-EGFP and endogenous TFEB together), or β-actin. Arrows at left and right sides of each panel indicate fast-migrating forms of TFEB-EGFP and TFEB proteins in chemical treated samples, compared with untreated samples (0 h). (**D**–**F**) Immunoblot analysis of immunoprecipitated TFEB-EGFP and 14-3-3 in MEF-TFEB-EGFP cells treated with translation inhibitors. The cells were treated with CHX (50 μg/mL) (**D**), LTM (500 nM) (**E**), or RocA (3 μM) (**F**) for indicated time and Torin2 (mTOR inhibitor, 100 nM) for 3 h as a positive control. Cells were lysed and subjected to immunoprecipitation with anti-GFP antibody. Immunoprecipitates were analyzed by immunoblotting with antibodies against GFP (for detecting TFEB-EGFP) and phospho-(Ser) 14-3-3 binding motif (known to bind phosphorylated TFEB-EGFP at Ser 211), or 14-3-3.

It has been proposed that the cellular localization and activity of TFEB are mainly controlled by its phosphorylation status [23,29,31] although other phosphorylation-independent mechanisms (such as SUMOylation, acetylation, and glucosylation) have also been reported [67,68,69,70]. Multiple studies have reported that TFEB migration on the protein gel is affected by its phosphorylation status [29,34,42]. Therefore, to assess the TFEB phosphorylation level indirectly, we analyzed changes in the electrophoretic mobility of both TFEB-EGFP and endogenous TFEB in CHX-, LTM-, RocA-, or Torin2-treated MEF-TFEB-EGFP cells. Torin2 treatment resulted in a rapid downshift in both TFEB-EGFP and TFEB migration. They were predominantly found in a fast-migrating form(s) in MEF-TFEB-EGFP cell lysates (Figure 2A). Treatment with CHX, LTM, or RocA led to a progressive shift in both TFEB-EGFP and endogenous TFEB bands to fast-migrating forms (arrows at left and right sides of the figures) in cell lysates under increased treatment times (Figure 2A–C). During the last time point (16 h) of inhibitor treatment, the fast-migrating TFEB-EGFP and endogenous TFEB bands (arrows) appeared to carry similar molecular weight compared with bands of Torin2 treatment, suggesting that CHX, LTM, and RocA induced TFEB activation via dephosphorylation, an effect analogous to that caused by Torin1 [29,42]. To substantiate these observations, we investigated whether TFEB phosphorylation at a specific site was affected by treatment with CHX, LTM, or RocA. Among 11 known phosphorylation sites of human TFEB [31,71], under normal nutrient conditions, mTORC1 phosphorylates TFEB at serine 211. This phosphorylation promotes the binding of TFEB to the chaperone 14-3-3 and retention of the transcription factor in the cytosol [23,31]. Therefore, we tested whether treatment with CHX, LTM, and RocA decreased the phosphorylation of TFEB-EGFP at Ser211 leading to the dissociation of 14-3-3 from the TFEB-EGFP/14-3-3 complex in MEF-TFEB-EGFP cells. TFEB-EGFP was immunoprecipitated with an anti-GFP antibody. TFEB-EGFP immunoprecipitates were used to determine the phosphorylation level of TFEB-EGFP at Ser211 and the TFEB-EGFP/14-3-3 complex. As expected, band intensities of p-TFEB-EGFP at Ser211 identified by immunoblotting with anti-phospho-(Ser) 14-3-3 binding motif antibody in TFEB-EGFP immunoprecipitates was markedly reduced by treatment with CHX, LTM, or RocA although these changes were not stronger than those induced by Torin2 (Figure 2D–F). Consistently, the band intensities of 14-3-3 proteins identified by immunoblotting with anti-14-3-3 antibody in TFEB immunoprecipitates were also markedly reduced by CHX, LTM, or RocA treatment, although these changes were not stronger than those induced by Torin2 (Figure 2D–F). Thus, the increased nuclear translocation of TFEB and TFE3 illustrated in Figure 1 and Figure 2 was likely due to dephosphorylation and dissociation of the 14-3-3 from the TFEB (or TFE3)/14-3-3 complex upon treatment with translation inhibitors.

### 2.3. Translation Inhibition-Mediated TFEB Nuclear Localization Is Modulated by Calcineurin, Not mTORC1

**Figure 3:**
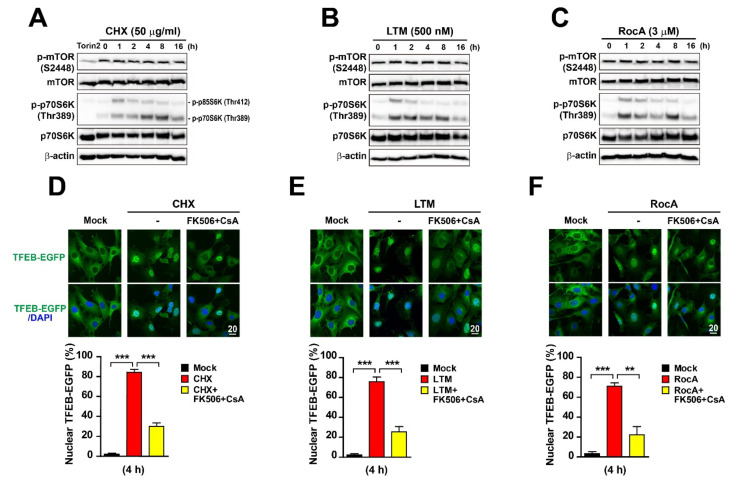
Calcineurin inhibition prevents translation inhibitor-induced TFEB nuclear translocation. (**A**–**C**) Immunoblot analysis of protein lysates obtained from MEF-TFEB-EGFP cells treated with CHX (50 μg/mL) (**A**), LTM (500 nM) (**B**), or RocA (3 μM) (**C**) for indicated time and Torin2 (mTOR inhibitor, 250 nM) for 3 h as a positive control. Total cellular lysates were analyzed by immunoblotting with indicated antibodies. (**D**–**F**) Representative images of confocal microscopic analysis. MEF-TFEB-EGFP cells were treated with Mock (DMSO), CHX (50 μg/mL) (**D**), LTM (500 nM) (**E**), or RocA (3 μM) (**F**) with or without calcineurin inhibitors [FK506 (10 μM) and cyclosporin A (CsA, 20 μM)] for 4 h. Cellular localization of TFEB-EGFP was indicated by green fluorescence in cells. DAPI (blue) staining indicates nucleus in merged images (lower panels). Scale bar, 20 μm. Graphs (lower panels) represent quantified results of the percentage of MEF-TFEB-EGFP cells with nuclear TFEB-EGFP upon chemical treatment. Data are expressed as mean ± SEM of at least 150 cells from 6 random fields in each group. ** *p* < 0.01 and *** *p* < 0.001; Mock vs. translation inhibitors or translation inhibitors vs. translation inhibitors + FK506 + CsA.

The phosphorylation status of TFEB is regulated by multiple kinases including mTORC1 and phosphatase calcineurin [23,31,38,42,71]. As shown in Figure 3D–F, inhibitor treatments reduced TFEB phosphorylation at serine 211, a target site of mTORC1 [5,33,72]. Therefore, we first investigated mTOR activation and its level. However, levels of mTOR phosphorylation (S2448) and mTOR were unchanged at all time points in cell lysates exposed to translation inhibitors, whereas the Torin2 treatment strongly reduced mTOR phosphorylation (Figure 3A–C). By contrast, phosphorylation (T389) of the mTOR target p70/S6 kinase was increased by CHX, LTM, or RocA treatment at all time points, whereas its phosphorylation was drastically inhibited by Torin2 treatment (Figure 3A–C). These results suggest that dephosphorylation-mediated TFEB and TFE3 nuclear translocation in cells treated with translation inhibitors might not involve mTOR. Next, we assessed whether inhibition of the phosphatase calcineurin can block TFEB nuclear translocation in translation inhibitor-treated MEF-TFEB-EGFP cells. For this, cells treated with CHX, LTM, or RocA were co-treated with both calcineurin inhibitors FK506 and cyclosporin A (CsA) for 4 h. As seen in Figure 3D–F, the translocation of TFEB-EGFP to the nucleus upon treatment with translation inhibitors was significantly reduced in the presence of calcineurin inhibitors, indicating that calcineurin may play an important role in the regulation of TFEB dephosphorylation and nuclear translocation in response to translation inhibition. However, it is important to note that inhibition of calcineurin did not completely abrogate the nuclear translocation of TFEB although it was significantly reduced by calcineurin inhibitors. Therefore, additional and unidentified factors might mediate TFEB dephosphorylation and nuclear translocation following treatment with translation inhibitors.

### 2.4. Translation Inhibition-Mediated TFEB Nuclear Localization Stimulates Autophagy-Related Gene Expression and Autophagosome Formation without New Protein Synthesis

**Figure 4:**
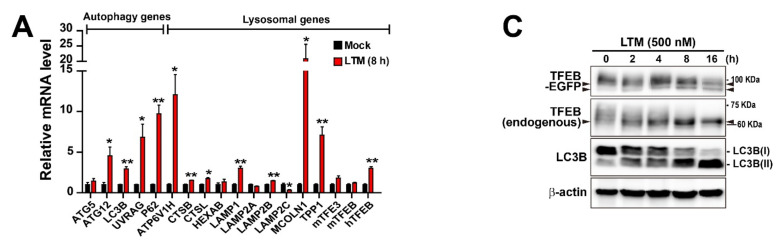

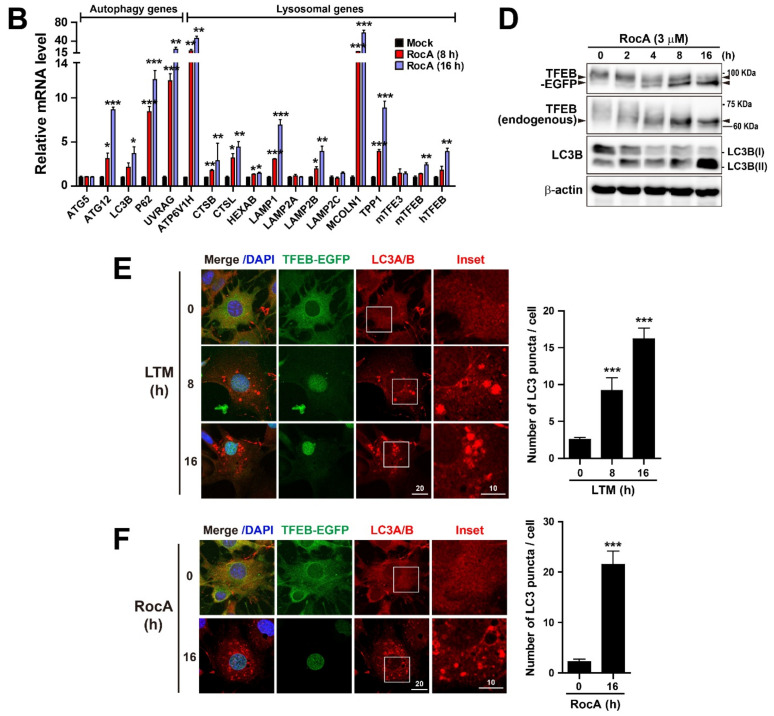
Translation inhibition induces autophagy-related gene expression and promotes autophagosome formation. (**A**,**B**) Quantitative RT-PCR analysis of mRNA expression of autophagy and lysosomal genes in MEF-TFEB-EGFP cells treated with LTM (Mock and 500 nM for 8 h) (**A**) or RocA (Mock and 3 μM for 8 h and 16 h) (**B**). Data are expressed as mean ± SEM from three independent experiments. * *p* < 0.05, ** *p* < 0.01, *** *p* < 0.001; Mock vs. Translation inhibitors. (**C**,**D**) Immunoblot analysis of protein lysates derived from MEF-TFEB-EGFP cells treated with LTM (500 nM) (**C**) or RocA (3 μM) (**D**) for indicated times. Total cellular lysates were analyzed by immunoblotting with antibodies against GFP, TFEB, LC3B, or β-actin. Arrows at left and right sides of each panel indicate fast-migrating forms of TFEB-EGFP and TFEB proteins in chemical treated samples, compared with untreated samples (0 h). (**E**,**F**) Immunofluorescence analysis of LC3/B-positive autophagosomes in MEF-TFEB-EGFP cells treated with LTM (500 nM for 0 h, 8 h, or 16 h) (**E**) or RocA (3 μM for 0 or 16 h) (**F**). Cells were fixed and stained with anti-LC3A/B (red). DAPI (blue) staining indicates nucleus in the merged images (the first column). Cellular localization of TFEB-EGFP is indicated by green fluorescence in the cells. Insets show a magnified view of the area outlined in the white lined box, Scale bar, 20 μm. Graphs represent results of quantification of LC3A/B puncta in each cell in left panel images. Data are expressed as mean ± SEM of at least 30 cells derived from 6 random fields in each group. *** *p* < 0.001; 0 h vs. 8 h or 16 h.

We next assessed whether LTM and RocA-induced TFEB/TFE3 nuclear translocation upregulated the transcription of autophagy-related genes. MEF-TFEB-EGFP cells were treated with LTM for 8 h or RocA for 8 and 16 h. Following 8 h of treatment, both inhibitors induced nuclear translocation of more than 80% TFEB and drastic translation inhibition (Figure 1B,C,E,F). Under the same conditions, LTM and RocA significantly increased the transcription of several autophagy genes and lysosomal genes downstream of TFEB/TFE3 in MEF-TFEB-EGFP cells (Figure 4A,B), suggesting that translation inhibition induces transcription of autophagy-related genes without new protein synthesis.

Although we observed a significant transcriptional increase in translation inhibition-mediated autophagy-related genes in LTM and RocA-treated cells, the results did not indicate activation of autophagic pathways, such as autophagosome formation. Therefore, to assess whether translation inhibition can promote autophagy, an autophagosome marker protein microtubule-associated proteins 1 light chain 3B (hereafter referred to as LC3B) was analyzed. The conversion of LC3B(I) to LC3B(II), a key molecule involved in autophagosome, is a key event in autophagosome formation [6]. As shown in Figure 4C,D, in line with dephosphorylation (arrows at left and right sides of the figures) of TFEB and TFEB-EGFP, following conversion of LC3B(I) to LC3B(II) in cells treated with LTM or RocA, levels of LC3B(II) were significantly increased in a time-dependent manner. Further, to directly monitor the possible impact of translation inhibitors on autophagosome formation, we visualized the presence of LC3A/B-positive puncta in LTM or RocA-treated cells (Figure 4E,F). As shown in Figure 4E,F, a marked increase in the number of LC3A/B-positive autophagic puncta in cells treated with LTM or RocA was observed. Together, our results indicate that translation inhibition may not only activate TFEB/TFE3, but also may stimulate autophagosome formation without new protein synthesis.

### 2.5. Translation Inhibitors Prevent Degradative Autolysosome Formation

Increased levels of LC3B(II) proteins and LC3A/B-positive autophagosomes following LTM and RocA treatment represent either autophagy activation or suppression of late steps in the autophagy pathway [5,72]. To ascertain whether the changes were caused by an increasing autophagic flux, we investigated the LC3B(II) and p62(SQSTM1) accumulation in cells incubated with bafilomycin A1 (Baf A1, 200 nM for 3 h before harvest), a specific inhibitor of vacuolar H^+^-ATPases and a blocker of autophagosome-lysosome fusion [18,72]. During autophagic flux, active LC3B(II) and p62 proteins accumulate upon Baf A1 treatment [73]. In the absence of treatment with translation inhibitors, the levels of both LC3(II) and p62 proteins were increased by Baf A1 as expected (Supplemental Figure S3A,B, lanes 1 vs. 4), indicating an active autophagic flux. However, with LTM treatment, Baf A1 failed to induce LC3B(II) and p62 accumulation (Supplemental Figure S4A, lanes 2, 3 vs. 5, 6), whereas RocA treatment induced their accumulation except for LC3B(II) at 16 h (Supplemental Figure S4B, lanes 2, 3 vs. 5, 6). Thus, the autophagic flux assay using Baf A1 did not clearly indicate whether LTM or RocA-induced translation inhibition increased autophagic flux. In addition, it is possible that strong translation inhibition and ubiquitin-proteasome pathway-mediated degradation [74,75] may prevent accumulation of both LC3(II) and p62 proteins in our assay conditions using the combination of both translation inhibitors and Baf A1.

Therefore, we next performed a different autophagic flux assay using an mRFP-GFP-LC3 tandem fluorescent probe [72,73]. HeLa cells were transfected with the mRFP-EGFP-LC3 construct and then treated with indicated compounds. At first, LTM and RocA-mediated translation inhibition was monitored by immunofluorescence of puromycylated proteins. A tandem fluorescent-tagged reporter was then used to monitor the autophagic flux, including the autophagosome synthesis and autophagosome-lysosome fusion. Autophagosomes appeared yellow (with green and red) and autolysosomes appeared as red vesicles since EGFP was quenched in acidic environments. However, mRFP is relatively stable. It can show fluoresce even in an acidic pH found in lysosomes [76,77]. As expected, Torin2 treatment increased the number of autolysosomes (mRFP^+^EGFP^−^ LC3B puncta) but reduced autophagosomes (mRFP^+^EGFP^+^ LC3B puncta) (left panels and graph in Supplemental Figure S4C), suggesting that the autophagic flux was significantly activated by mTORC1 inhibition. However, following LTM or RocA treatment, which induced strong translation inhibition at 8 and 16 h (puromycin of Supplemental Figure S4C), the number of autolysosomes (mRFP^+^EGFP^−^ LC3B puncta) was not increased although the number of autophagosomes (mRFP^+^EGFP^+^ LC3B puncta) was increased (right panels and graphs of Supplemental Figure S4C), indicating possible defects in the autophagic flux.

**Figure 5:**
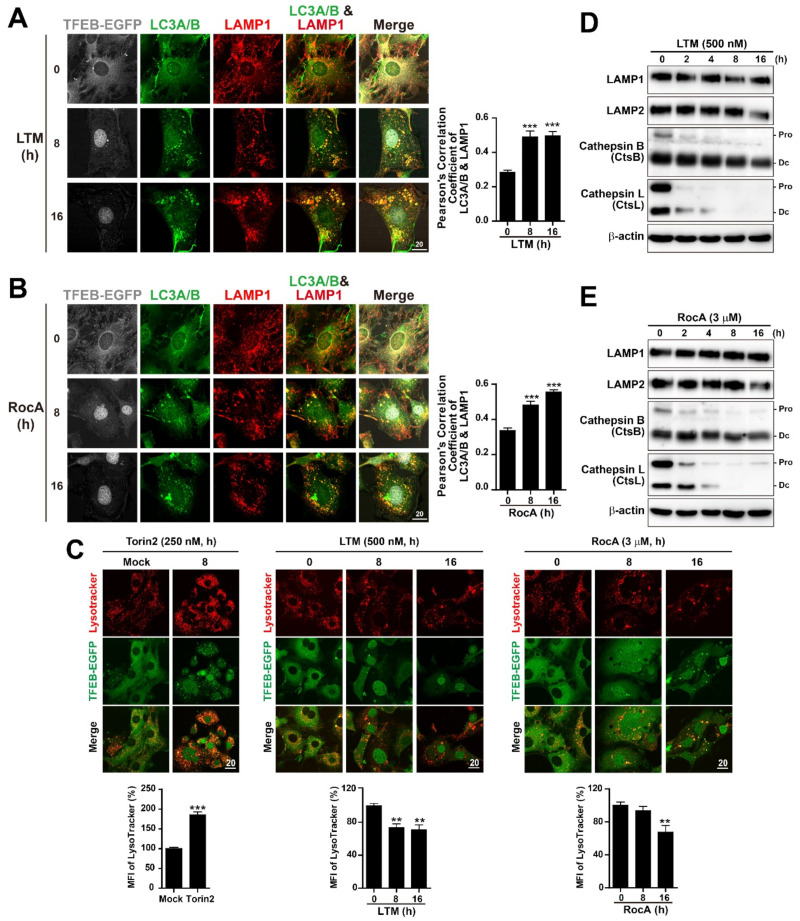
Translation inhibition induces autolysosome formation but lysosomal dysfunction. (**A**,**B**) MEF-TFEB-EGFP cells were treated with LTM (500 nM) (**A**) or RocA (3 μM) (**B**) for the indicated time. Cells were fixed and co-stained with anti-LC3A/B (green) and anti-LAMP1 (red). Green fluorescence of TFEB-EGFP was converted to gray. Scale bar, 20 μm. Graphs represent results of quantification of LC3A/B and LAMP1-double positive puncta per cell in the left panel of images. Data are expressed as mean ± SEM of at least 30 cells from 6 random fields in each group. *** *p* < 0.001; 0 h vs. 8 h or 16 h. (**C**) MEF-TFEB-EGFP cells were treated with LTM (500 nM) or RocA (3 μM) for indicated times and Torin2 (mTOR inhibitor, 250 nM) for 8 h as a positive control. Acidic vesicles were visualized with LysoTracker Red (100 nM, 15 min) (red) and cellular localization of TFEB-EGFP is indicated by green fluorescence signal in cells. Scale bar, 20 μm. Mean fluorescent intensity (MFI) of LysoTracker Red was quantified and presented in the graphs. Data are expressed as mean ± SEM of at least 30 cells derived from 6 random fields in each group. ** *p* < 0.01 and *** *p* < 0.001; 0 h vs. 8 h or 16 h. (**D**,**E**) Immunoblot analysis of protein lysates from MEF-TFEB-EGFP cells treated with LTM (500 nM) (**C**) or RocA (3 μM) (**D**) for indicated times. The total cellular lysates were analyzed by immunoblotting with indicated antibodies. Pro: procathepsin form. Dc: heavy chain of the double-chain form.

To determine which step in the autophagic flux was inhibited upon treatment with LTM or RocA, we first evaluated the fusion of autophagosomes with lysosomes, which is an important stage in the autophagic flux. Autophagosome-lysosome fusion can be detected via colocalization of LC3 and lysosomal-associated membrane protein 1 (LAMP1) [5,72]. We performed immunostaining of LC3A/B and LAMP1 and quantified the colocalization of LC3A/B and LAMP1 in LTM- or RocA-treated MEF-TFEB-EGFP cells. However, intriguingly, we found that the colocalization of LC3A/B and LAMP1 was significantly increased in LTM- or RocA-treated cells following the nuclear translocation of TFEB-EGFP when incubation time was increased (Figure 5A,B), indicating the fusion of autophagosomes with endosomes and/or lysosomes at least. However, the late steps of the autophagic process to maintain the functional autophagic flux includes autolysosome acidification as well as autophagosome-lysosome fusion to form degradative autolysosomes [5,18]. Autolysosome acidification requires active lysosomes independent of autophagosome-lysosome fusion [18]. In addition, the results of our autophagic flux assay using tandem fluorescent-tagged LC3 (mRFP-EGFP-LC3) suggested that LTM or RocA treatment did not increase the number of acidic autolysosomes (Supplemental Figure S4C). Therefore, we asked whether LTM or RocA treatment affected lysosomal acidification and lysosomal protein levels. In Torin2-treated cells, the induction of autophagy resulted in the nuclear translocation of TFEB-EGFP and increased autophagic vesicles strongly labeled with LysoTracker, a dye known to accumulate in acidic vesicles (Figure 5C and Figure S4D). However, LTM or RocA treatment significantly reduced the number and intensity of LysoTracker-positive structures in both MEF-TFEB-EGFP cells (Figure 5C) and HeLa cells (Supplementary Figure S4D), suggesting that the translation inhibition disrupted lysosomal acidity and reduced the number of functional lysosomes. Further, we found that LTM or RocA treatment strongly reduced the expression of cysteine cathepsins B and L (CtsB/L) known to be major lysosomal proteases, whereas LAMP1 and LAMP2 as major protein constituents of the lysosomal membrane, were not significantly changed (Figure 5D,E). Thus, these findings suggest that LTM- or RocA-mediated translation inhibition induces autophagic vesicle (AV) formation but prevents degradative autolysosome formation.

**Figure 6:**
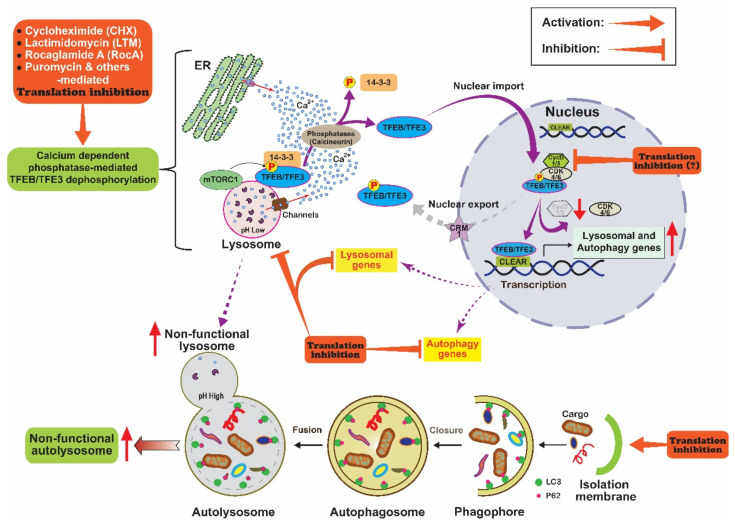
Model to explain translation inhibition-mediated regulation of TFEB/TFE3 nuclear localization and autophagy. Chemical translation inhibitors (CHX, LTM, puromycin and others)-mediated translation inhibition may induce cytosolic release of Ca^2+^ from the ER or lysosome [78,79]. Higher cytosolic Ca^2+^ concentration then activates phosphatases including calcineurin, which can then dephosphorylate TFEB and TFE3 [38,40,80,81]. These dephosphorylated TFEB and TFE3 are released from 14-3-3 proteins and can translocate to the nucleus [33,35,36,37]. Under normal conditions, CDK4/6 are activated by cyclin Ds in the nucleus. These activated kinases can then interact with and phosphorylate TFEB and TFE3, promoting their CRM1-dependent nuclear export and inactivation [32]. However, CDK4/6 might be inactivated owing to reduced levels of cyclin Ds when translation is inhibited. TFEB and TFE3 are not phosphorylated by CDK4/6 and thus are retained in the nucleus, where they can induce transcription of lysosomal and autophagy genes. In addition, translation inhibition can promote autophagosome formation and autophagosome-lysosome fusion. However, it ends up inducing lysosomal dysfunction and preventing degradative autolysosome formation owing to protein synthesis inhibition on lysosomal and autophagy gene expression.

Here we analyzed the effects of three different translation inhibitors on the activation of the autophagy master transcriptional regulators TFEB/TFE3 and autophagic pathways. The translation inhibitors induced the nuclear translocation of both TFEB and TFE3 in human and mouse cells. Their translocation was related to translation inhibition-mediated dephosphorylation and 14-3-3 dissociation. However, TFEB dephosphorylation did not occur via mTORC1 inhibition, but via activation of calcineurin and/or other phosphatases in cells treated with translation inhibitors. Surprisingly, LTM and RocA strongly stimulated the expression of several _autophagic_ and lysosomal genes without a new protein synthesis. In addition, we observed that LTM and RocA promoted autophagosome formation. However, the translation inhibition prevented the degradative autolysosome formation and induced lysosomal dysfunction. Therefore, translation inhibition might be one of the components of TFEB/TFE3 activation although it eventually induces dysfunction of autophagosome maturation probably because of inhibition of protein synthesis (Figure 6).

## 3. Discussion

More than 370 inhibitors of eukaryotic protein synthesis have been reported [47]. Using three translation inhibitors (CHX, LTM, and RocA) with distinct inhibitory mechanisms, we analyzed their effects on the regulation of TFEB activity and autophagy. All three translation inhibitors acted similarly on TFEB and TFE3 activation and autophagic processes, suggesting activation of TFEB and TFE3 via common molecular mechanisms. Inhibitor treatments triggered TFEB nuclear translocation at 1 h when translation inhibition was less than 60% (Figure 1D–F), indicating a rapid molecular mechanism(s) without new protein synthesis. We thought that calcium, a ubiquitous second messenger, was a candidate mediator because its level can be rapidly changed to regulate the activities of multiple enzymes including calcineurin phosphatase [82]. In addition, the activation of calcineurin can promote the nuclear translocation of TFEB and TFE3 [38,80,81]. As expected, our results showed that calcineurin inhibitors (FK506 and CsA) reduced translation inhibitor-mediated TFEB nuclear translocation, although their inhibitory effect was not complete, whereas no mTOR inhibition was observed (Figure 4A–F). However, the mechanism of the activation of calcium-dependent calcineurin upon translation inhibition is unknown. Several studies have suggested that the ER, lysosome, and mitochondria are sources of calcium, which can activate phosphatases including calcineurin responsible for TFEB/TFE3 activation [38,40,80,81]. Interestingly, previous studies have reported that puromycin, another translation inhibitor, can inhibit peptidyl transfer on ribosomes, causing ER calcium leakage via Sec61 translocon [78,79]. In addition, we observed that puromycin also promoted the nuclear translocation of TFEB [83]. Overall, these findings suggest that calcium is an important mediator of translation inhibition-mediated TFEB/TFE3 activation (Figure 6). However, further studies are needed to determine the cellular organelles and molecular mechanism involved in calcium release and to identify phosphatases including calcineurin responsible for TFEB/TFE3 activation upon translation inhibition.

Recent studies have suggested that subcellular localization of TFEB can be determined via regulation of the TFEB nuclear export, because it continuously shuttles between the cytosol and nucleus via the CRM1-dependent nuclear export under normal conditions [29,30,32]. Among those studies, Yin et al. [32] suggested that cyclin D-dependent kinases 4/6 (CDK4/6) can interact with and phosphorylate TFEB on serine 142 and TFE3 on serine 246 in the nucleus, resulting in their inactivation and cytoplasmic translocation (Figure 6). Therefore, CDK4/6 inhibition can induce TFEB/TFE3 nuclear translocation, thereby activating TFEB/TFE3-dependent autophagic pathways including autophagic gene expression and lysosome biogenesis [32]. Interestingly, multiple reports have shown that CHX treatment can reduce the expression of cyclin D1 [84,85,86], a well-known cell cycle regulator which dimerizes with CDK4/6 and facilitates to pass through the G1 phase by inhibiting the retinoblastoma protein [87,88]. The cyclin D level can be reduced by inhibiting its transcription and translation, and even inducing proteasome- and autophagy-mediated degradation [84,85,89,90]. However, under our experimental conditions, translational inhibition and proteasome-mediated degradation were the most plausible mechanisms underlying decreased cyclin D because the inhibition of protein synthesis disrupted the last step (degradative autolysosome formation) of autophagy (Figure 5C–E and Figure S4C,D) and pre-emptively nullified the possible effect of reduced cyclin D1 transcription. Therefore, it is possible that translation inhibition-mediated cyclin D reduction inhibits CDK4/6 activity in the nucleus, thereby activating TFEB/TFE3 (Figure 6). These possible mechanisms are under investigation now.

In previous studies investigating the effect of CHX pre-treatment on autophagic pathways, no nascent autophagic vesicles has been found to be cathepsin-D positive, suggesting that the CHX-mediated inhibition of protein synthesis does not inhibit the initial formation of autophagosomes, but interferes with a later stage in autophagosome maturation such as autophagosome-lysosome fusion [60]. In this study, we observed that LTM and RocA treatments increased LC3B(II) protein level and LC3A/B-positive autophagic puncta (Figure 4C–F, Figure 5A,B and Figure S4A–C), indicating that LTM and RocA activated the formation of autophagosomes. However, similar to the CHX effect [59], LTM or RocA-mediated translation inhibition impaired the autophagic flux (Supplementary Figure S4A–D). LTM or RocA treatment resulted in an increase in LC3A/B and LAMP1-double positive autophagic puncta (Figure 5A,B), without increasing mRFP positive and EGFP negative LC3B autophagic puncta (Supplementary Figure S4C,D), indicating that LTM and RocA-mediated translation inhibition prevented degradative autolysosome formation in autophagosome maturation. Accordingly, treatment with LTM and RocA reduced the number of functional lysosomes by disrupting lysosomal acidity (Figure 5C) and substantially reducing the expression of major lysosomal proteases CtsB and CtsL (Figure 5D,E). In addition, Mauvezin et al. suggested that, although non-functional lysosomes such as V-ATPase-deficient lysosomes can fuse with autophagosomes, they prevent degradative autolysosome formation [18]. Therefore, it is possible to generate non-functional autolysosomes via fusion between autophagosomes and non-functional lysosomes in LTM or RocA-treated cells (Figure 6).

## 4. Materials and Methods

### 4.1. Antibodies and Reagents

The following antibodies were used in this study: rabbit polyclonal anti-GFP (A-11122, Invitrogen, Carlsbad, CA, USA); mouse monoclonal anti-GFP (B-2) (sc-9996), mouse monoclonal anti-pan 14-3-3 (sc-133232) (Santa Cruz Biotechnology, Dallas, TX, USA), rabbit polyclonal anti-TFEB (A303-673A, Bethyl Laboratories, Montgomery, TX, USA), rabbit polyclonal anti-TFEB (MBS9125929, MyBioSource, San Diego, CA, USA), mouse monoclonal anti-phospho-(Ser) 14-3-3 binding motif (#9606), rabbit polyclonal anti-LC3A/B (#4108), rabbit monoclonal anti-mTOR (#2983), rabbit polyclonal anti-phosphoSer2448-mTOR (#2971), rabbit polyclonal anti-p70 S6 kinase (#2971), mouse monoclonal anti-phospho Thr389-p70 S6 kinase (#9206) (Cell Signaling Technology, Danvers, MA, USA), rabbit polyclonal anti-LC3B (L7543), rat monoclonal anti-LAMP1 (1D4B-C), rat monoclonal anti-LAMP2 (ABL-93-C) (Developmental Studies Hybridoma Bank, Iowa City, IA, USA), goat polyclonal anti-Cathepsin B (AF965), goat polyclonal anti-Cathepsin L (AF1515) (R&D Systems, Minneapolis, MN, USA), mouse monoclonal anti-β-Actin (A5441) (Sigma-Aldrich, St. Louis, MO, USA), rabbit polyclonal anti-histone H3 (ab1791, Abcam, Cambridge, UK), mouse monoclonal anti-puromycin (MABE343, Merck Millipore, Burlington, MA, USA), peroxidase-conjugated AffiniPure goat anti-rabbit IgG (H + L) (111-035-003), peroxidase-conjugated AffiniPure F(ab’)_2_ fragment donkey anti-mouse IgG H + L) (751-036-151), peroxidase-conjugated AffiniPure rabbit anti-goat IgG (H + L) (305-035-003), peroxidase AffiniPure goat anti-rat IgG (H + L) (112-035-003), Alexa Fluor 594-conjugated AffiniPure F(ab’)_2_ fragment donkey anti-rabbit IgG (H + L) (711-586-152), Alexa Fluor 594-conjugated AffiniPure F(ab’)_2_ fragment goat anti-mouse IgG (H + L) (115-586-003), Alexa Fluor 647 AffiniPure F(ab’)_2_ fragment goat anti-mouse IgG (H + L) (115-606-146) (Jackson Immuno Research Laboratories, Inc, West Grove, PA, USA), goat anti-rabbit Alexa Fluor 488 (A-11034), and goat anti-rat Alexa Flour 647 (A-21247) (Invitrogen, Carlsbad, CA, USA). Rabbit polyclonal anti-TFE3 was a gift from Professor Hiderou Yoshida, Department of Molecular Biochemistry, Graduate school of Life Science, University of Hyogo, Hyogo, Japan.

The following chemicals were used in this study: cycloheximide (CHX, C-7698, Sigma-Aldrich), rocaglamide A (RocA, 14841) and bafilomycin A1 (Baf A1, 11038) (Cayman Chemical, Michigan, USA), lactimidomycin (LTM, 5.06291.0001, Merck Millipore, Burlington, MA, USA), LysoTracker Red DND-99 (L7528, Thermo Fisher Scientific, Waltham, MA, USA), puromycin (sc-108071) and cyclosporin A (CsA, sc-3503) (Santa Cruz Biotechnology, Dallas, TX, USA), Torin2 (4248, Tocris Bioscience, Bristol, UK), FK506 (Tlrl-FK5, InvivoGen, San Diego, CA, USA), 4′,6-diamidino-2-phenylindole dihydrochloride (DAPI, D1306), and blasticidin S HCl (R210-01) (Invitrogen, Carlsbad, CA, USA). 

### 4.2. Construction of Expression Plasmids

For TFEB-EGFP or EGFP expression, human TFEB fused with the enhanced green fluorescent protein (EGFP) expressing pLUB-hTFEB-EGFP-IRES-Bla or EGFP expressing pLUB-EGFP-IRES-Bla plasmids was constructed. The pLUB-IRES-Bla plasmid was constructed by changing the CMV promoter of pLVX-IRES-Bla with the ubiquitin C promoter of pUB-EGFP (11155, Addgene, Watertown, MA, USA). The Ssp1-EcoRI fragment containing ubiquitin C promoter from pUB-EGFP was inserted into pLVX-IRES-Bla treated with ClaI-Klenow-EcoRI. To construct pLUB-hTFEB-EGFP-IRES-Bla, the NotI-Klenow-EcoRI fragment containing hTFEB-EGFP from pEGFP-N1-TFEB (38119, Addgene, Watertown, MA, USA) was inserted into pLUB-IRES-Bla treated with SmaI-EcoRI. To construct pLUB-EGFP-IRES-Bla, the NotI-Klenow-EcoRI fragment containing EGFP of pEGFP-N1 (#6085-1, Clontech, Takara Bio USA, Inc., San Jose, CA, USA) was inserted into pLUB-IRES-Bla treated with BamHI-Klenow-EcoRI.

To generate a tandem fluorescent reporter expressing mRFP-EGFP-LC3B, pEGFP-LC3B was constructed by inserting the cDNA fragment encoding LC3B from pmRFP-LC3B (21075, Addgene, Watertown, MA, USA) treated with BglII and BamHI into the pEGFP-C1 (6084-1, Clontech Laboratories, San Jose, CA, USA) treated with the same restriction enzymes. The coding sequence fragment of mRFP was amplified from pmRFP-LC3B vector via polymerase chain reaction (PCR) with the following primers: 5′-GAGAGCTAGCGGCCACCATGGCCTCCTCCGAGGAC-3′ and 5′-GAGAACCGGTCCACCGGCGCCGGTGGAGTGGCG-3′. The tandem fluorescent reporter pmRFP-EGFP-LC3B was constructed by inserting the PCR product of the mRFP sequence treated with NheI and AgeI into pEGFP-LC3B treated with the same restriction enzymes.

### 4.3. Establishment of Cell Culture and Cell Line

Mouse embryonic fibroblast (MEF) cells were described previously [91] and cultured in Dulbecco’s modified Eagle’s medium (DMEM) supplemented with 10% fetal bovine serum (FBS) (WelGENE, Gyongsan, Korea), 1% penicillin-streptomycin (WelGENE) and 1% non-essential amino acids (WelGENE), as previously described [91]. Immortalized hepatocytes were described previously [64] and grown in Medium 199 (WelGENE) supplemented with 10% FBS (WelGENE) and 1% penicillin-streptomycin (WelGENE). The HeLa cell line was purchased from Korean cell line bank and cultured in MEM Alpha medium (M0894, Sigma-Aldrich, St. Louis, MO, USA) supplemented with 4.4 mg/mL sodium bicarbonate, 10% FBS (WelGENE), and 1% penicillin-streptomycin (WelGENE). All cells were incubated at 37 °C with 5% CO_2_.

To generate TFEB-EGFP or EGFP stably expressing MEF cell lines, lentiviral particles containing pLUB-hTFEB-EGFP-IRES-Bla or pLUB-EGFP-IRES-Bla constructs were produced in Lenti-X-293T cells using LentiX packaging Single Shot Protocol-At-A-Glance Kit (Clontech). Lentivirus production was verified using Lenti-X GoStix™ (Takara Korea Biomedical Inc, Seoul, Korea). MEF cells were transduced with each virus supernatant for 48 h. These infected cells were layered at one cell per well into 96-well cell culture plates via serial dilutions. These cells were cultured in the culture media containing 5 μg/mL blasticidin S HCl (Invitrogen, Carlsbad, CA, USA) for 10 days. The expression of TFEB-EGFP or EGFP was monitored by western blot with anti-GFP antibody and anti-TFEB. Green fluorescence-positive colonies were examined under confocal microscopy using an FV1200-OSR microscope (Olympus, Shinjuku, Japan) as described in the next section. Among several positive clones identified, we selected those that showed high expression of TFEB-EGFP or EGFP and regulated nuclear translocation of TFEB-EGFP upon treatment with well-known autophagy inducers such as Earle’s balanced salt solution (EBSS) or Torin2.

### 4.4. Immunofluorescences Staining and Confocal Microscopy

Cells were plated on collagen-coated glass coverslips in 6-well dishes and cultured overnight. These cells were treated with the indicated chemicals for indicated times, followed by rinsing twice with phosphate-buffered saline (PBS). Cells were then fixed with 4% paraformaldehyde in PBS for 15 min, and permeabilized with 0.1% Triton X-100 in PBS for 5 min. For puromycin, TFEB or TFE3 staining, cells were blocked with 3% bovine serum albumin (BSA) in PBS for 1 h and incubated with the indicated primary antibodies overnight at 4 °C. Especially, for TFE3 staining in immortalized hepatocytes, cells were blocked with 2.5% normal horse serum (S-2012, Vector Laboratories, Burlingame, CA, USA) for 1 h and incubated with the primary antibody against TFE3 overnight at 4 °C.

To visualize LC3A/B puncta or the colocalization of punctate LC3A/B and LAMP1, the cells on the coverslip were fixed with 100% methanol for 10 min at −20 °C. These cells were blocked with 3% BSA in PBS containing 0.05% Tween-20 for 1 h and incubated with the indicated primary antibodies overnight at 4 °C.

Cells incubated with the indicated primary antibodies were further incubated with fluorescence-conjugated secondary antibodies for 1 h at room temperature, followed by nuclear staining with DAPI (Invitrogen). Finally, coverslips were mounted on ProLong Gold mounting medium (Invitrogen) and cells were visualized via confocal laser microscopy using a FV1200-OSR microscope (Olympus, Shinjuku, Japan). The puromycin intensity was measured using the mean fluorescence intensity tool in FV10-ASW-4.2 software (Olympus, Shinjuku, Japan). The colocalization of LC3A/B and LAMP1 was measured using Pearson’s correlation coefficient calculator tool in the FV10-ASW-4.2 software (Olympus, Shinjuku, Japan).

For lysosome staining, MEF TFEB-EGFP cells and HeLa cells were plated on collagen-coated 35-mm glass bottom confocal dishes (101,350, SPL Life Science, Pocheon-si, Gyeonggi-do, Korea) at a density of 1.2 × 10^5^ cells. On the next day, the cells were treated with the indicated chemicals in a phenol-red free culture medium (DMEM or MEM, GIBCO, Carlsbad, CA, USA) for the indicated times. In the last 30 min of the chemical treatment, 100 nM LysoTracker Red DND-99 (L7528) (Invitrogen, Carlsbad, CA, USA) was added to the cell culture media. The live images of lysosomes were visualized under an FV1200-OSR microscope (Olympus, Shinjuku, Japan). The LysoTracker Red intensity was measured using the mean fluorescence intensity tool in FV10-ASW-4.2 software (Olympus, Shinjuku, Japan).

For the autophagic flux assay using a tandem fluorescent probe, the pmRFP-EGFP-LC3B was transfected into the HeLa cells using Mirus Bio *Trans*IT-LT1 transfection reagent (MIR2306, Thermo Fisher Scientific, Waltham, MA, USA) according to the manufacturer’s instructions for 30 h, followed by treatment with Torin2, LTM or RocA for the indicated times. These cells were additionally incubated with puromycin (10 μg/mL for 10 min) to label actively translating peptides. Cells were then prepared as described above.

### 4.5. Immunoblot Analysis

Cells were lysed in Nondiet P40 lysis buffer (1% NP40, 50 mM Tris-Cl pH 7.5, 150 mM NaCl, 0.05% SDS, 0.5 mM Na-vanadate, 100 mM NaF, 50 mM β-glycerophosphate, and Halt Protease Inhibitor Cocktail (Thermo Fisher Scientific, Waltham, MA, USA). Cell lysates were centrifuged at 13,000× *g* for 15 min at 4 °C and supernatants were collected. The protein concentration was determined using Pierce™ BCA Protein Assay Kit (Thermo Fisher Scientific, Waltham, MA, USA). Next, the same amount of protein lysate was subjected to sodium dodecyl sulfate-polyacrylamide gel electrophoresis (SDS-PAGE), followed by transfer to polyvinylidene difluoride or nitrocellulose membranes. The membranes were blocked with 5% non-fat skim milk in 1× Tris-Buffered Saline-Tween 20 (0.1% Tween 20, 20 mM Tris-HCl, pH 7.5, and 150 mM NaCl) at room temperature for 1 h. Membranes were incubated with the indicated primary antibodies at 4 °C overnight and then with horseradish peroxidase-conjugated secondary antibodies for 1 h at room temperature. Targeted proteins were visualized with SuperSignal™ West Pico PLUS Chemiluminescent substrate (Thermo Fisher Scientific, Waltham, MA, USA) and detected using an Azure Biosystems C300 (Azure Biosystems, Inc., Dublin, CA, USA).

### 4.6. Co-Immunoprecipitation Assay

TFEB-EGFP expressing MEF (MEF-TFEB-EGFP) cells were plated onto 100 mm culture dishes at a density of 7 × 10^5^ cells. The next day, cells were treated with CHX, LTM or RocA for the indicated times. These cells were collected in completed growth media and then washed once with PBS. Cell pellets were dissolved in 250 μL immunoprecipitation (IP) lysis buffer (50 mM Tris HCl, pH 7.5, 150 mM NaCl, 1% Triton X-100, 1 mM EDTA) supplemented with half protease and phosphatase inhibitor cocktail at 1× final concentration (Thermo Fisher Scientific, Waltham, MA, USA). Lysed cells were passed through a 26 G needle eight times. Cell lysates were incubated on ice for 20 min and centrifuged at 13,000× *g* for 15 min at 4 °C to collect soluble fractions. To prepare the antibody-bead complex, 2 μg/mL of GFP antibody (Invitrogen, Carlsbad, CA, USA) was rotated with 30 μL of slurry containing protein A/G agarose plus beads (Thermo Fisher Scientific, Waltham, MA, USA) in 1 mL of IP binding buffer (50 mM Tris HCl, pH 7.5, 100 mM NaCl, 1 mM EDTA) supplemented with half protease and phosphatase inhibitor cocktail at 1× final concentration (Thermo Fisher Scientific, Waltham, MA, USA) for 3 h at 4 °C. Then, 1 mg protein lysate was diluted in 1 mL of the IP binding buffer and transferred to the GFP antibody-protein A/G agarose bead complex and incubated under rotation for an additional 3 h at 4 °C. After incubation, beads were washed twice in 1 mL of the IP binding buffer. Samples were eluted with 45 μL of 1.5× SDS sample loading buffer, boiled at 100 °C for 5 min, and separated by SDS-PAGE.

### 4.7. Subcellular Fractionation

The MEF-TFEB-EGFP cells were plated on 100 mm culture dishes at a density of 7 × 10^5^ cells. The next day, cells were treated with CHX, LTM, or RocA for the indicated times. Cells were collected in the completed growth media, followed by washing once with cold PBS. Cell pellets were lysed in ice-cold hypotonic buffer (10 mM HEPES, 10 mM KCl, 0.1 mM EDTA, 0.5% NP-40, 1 mM DTT, half protease and phosphatase inhibitor cocktail, pH 7.4) for 40 min on ice. Cell lysates were centrifuged at 13,000× *g* for 15 min. Supernatants were collected as cytoplasmic fractions. Cell pellets obtained as nuclear fractions were washed with the hypotonic buffer to completely remove the residual cytoplasmic fractions. The cell pellets were resuspended in nuclear extraction buffer (20 mM HEPES, 400 mM NaCl, 1 mM EDTA, 1 mM DTT, half protease and phosphatase inhibitor cocktail). The protein concentration was measured, and the lysate samples were analyzed using western blot.

### 4.8. RNA Isolation and Quantitative Real-Time Polymerase Chain Reaction (qRT-PCR) Analysis

The total RNAs were isolated from MEF-TFEB-EGFP cells treated with indicated chemicals for indicated time using a QIAzol lysis reagent (QIAGEN, Hilden, Germany). The cDNAs were synthesized with a high-capacity cDNA RT kit (Applied Biosystems, Waltham, CA, USA) for quantitative polymerase chain reaction (qPCR). The qPCRs were carried out with a TOPreal™ qPCR 2X PreMIX (SYBR Green with High ROX) (RT501M, Enzynomics, Daejeon, Korea) using a StepOnePlus Real Time System (Applied Biosystems, Waltham, CA, USA). The specificity of each primer pair was confirmed using the melting curve analysis. The copy number relative to β-actin mRNA was calculated as previously described [92]. Primer sequences are presented in Supplemental Table S1.

### 4.9. Statistical Analysis

All data are presented as means ± SEM. The data were analyzed using GraphPad Prism 5 (GraphPad Software, San Diego, CA, USA). The statistical significance of differences between groups was evaluated via the unpaired two-tailed Student’s *t*-tests and *p* < 0.05 was considered statistically significant.

## 5. Conclusions

In this study, we demonstrated the previously unrecognized function of a well-known translation inhibitor CHX and two new translation inhibitors, LTM and RocA, in autophagy, which can be used to destroy rapidly growing tumor cells and impair or delay the spread of fast-replicating viral pathogens. Further studies on autophagy regulation by these inhibitors might reveal more detailed molecular mechanisms of cell death induced by them, which can be used to develop cancer or antiviral drugs. In addition, as our findings suggest a new biological function of translation inhibition in autophagy regulation, it is worthwhile to find a cellular translation regulation factor that may share molecular mechanism(s) of TFEB/TFE3 activation with translation inhibitors. Such investigation may introduce a new cellular mechanism of translation inhibition-mediated autophagy regulation.

## Data Availability

Not applicable.

## Supplementary Materials

The following are available online at https://www.mdpi.com/article/10.3390/ijms222112083/s1.
