# Supplementary material for: Translation Inhibitors Activate Autophagy Master Regulators TFEB and TFE3
Source: Int J Mol Sci. 2021 Nov 8;22(21):12083. doi: 10.3390/ijms222112083 (PMC8584619; doi:10.3390/ijms222112083)
Supplement: Supplementary file 1 [file ijms-22-12083-s001.zip › ijms-1431503-supplementary.pdf]

Supplementary Figures

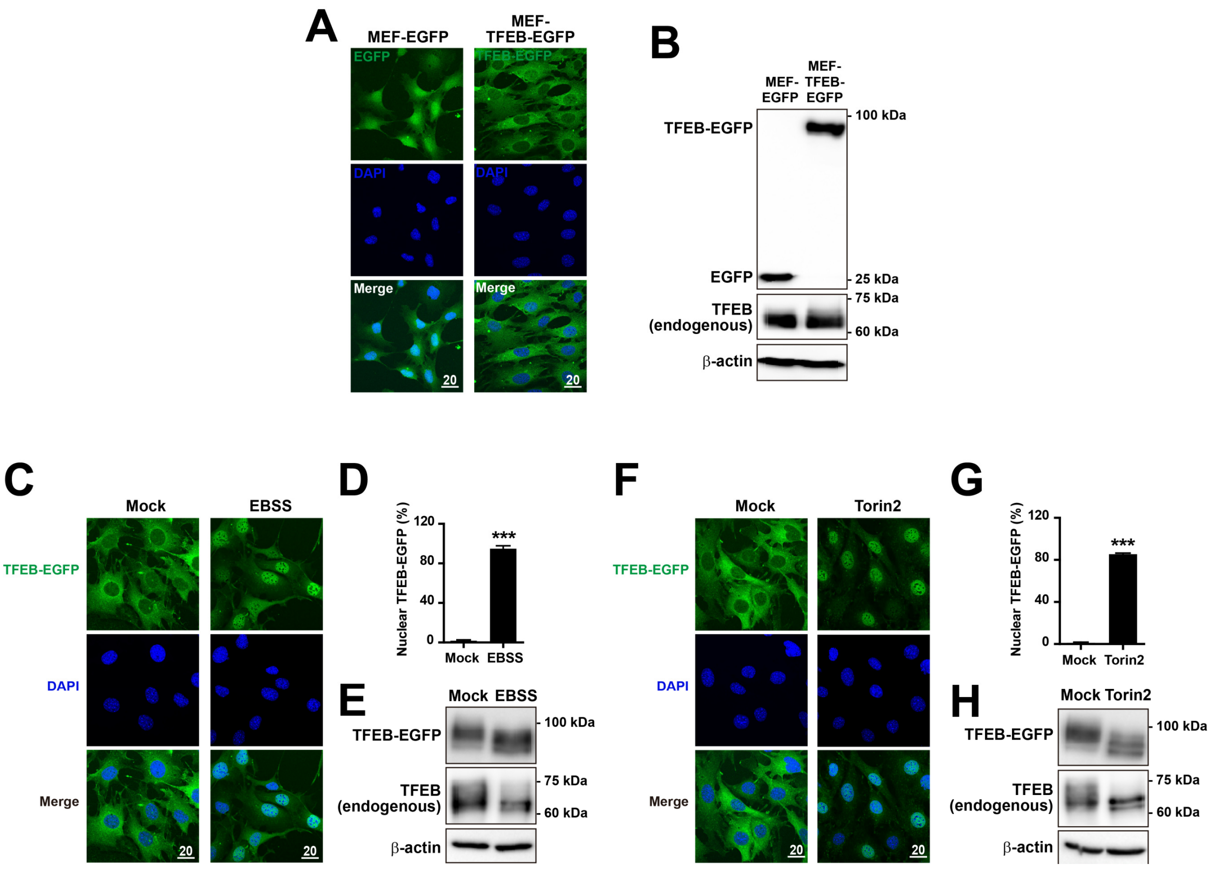

Figure S1. Establishment of an MEF cell line stably expressing TFEB-EGFP. **(A)** EGFP or TFEB-EGFP expressing MEF cells were transduced with lentiviral particles containing pLUB-EGFP-IRES-Bla or pLUB-hTFEB-EGFP-IRES-Bla constructs, followed by blasticidin selection. From the selected cell lines, cells were fixed and stained with DAPI (blue) for nucleus DNA. Cellular localization of EGFP or TFEB-EGFP was indicated by green fluorescence in the cells. Scale bar, 20 µm. **(B)** Immunoblot analysis of total cellular lysates from MEF cells stably expressing EGFP or TFEB-EGFP using the antibodies against GFP to detect TFEB-EGFP and EGFP combined, TFEB (used to detect endogenous TFEB), or β-actin. **(C,F)** Representative images of confocal microscopic analysis. TFEB-EGFP-expressing MEF (MEF-TFEB-EGFP) cells were starved with Earle's balanced salt solution (EBSS) (C) or treated with Torin2 (100 nM) (F) for 4 h. Cells were fixed and stained with DAPI (blue) for DNA. Cellular localization of TFEB-EGFP was indicated by green fluorescence signals in the cells. Scale bar, 20 µm. **(D,G)** Quantification of the percentage of MEF-TFEB-EGFP cells with nuclear TFEB-EGFP treated with EBSS or Torin2 as indicated in (C) and (F). Data are expressed as mean ± SEM of at least 150 cells from six random fields in each group. ***$p < 0.001$; Mock vs. EBSS or Torin2. **(E,H)** MEF-TFEB-EGFP cells were treated with EBSS (C), Torin2 (F) for 4 h. Total cellular lysates were separated via 6% SDS-PAGE and then analyzed by immunoblotting with antibodies against GFP (for TFEB-EGFP), TFEB (for endogenous TFEB), or β-actin.

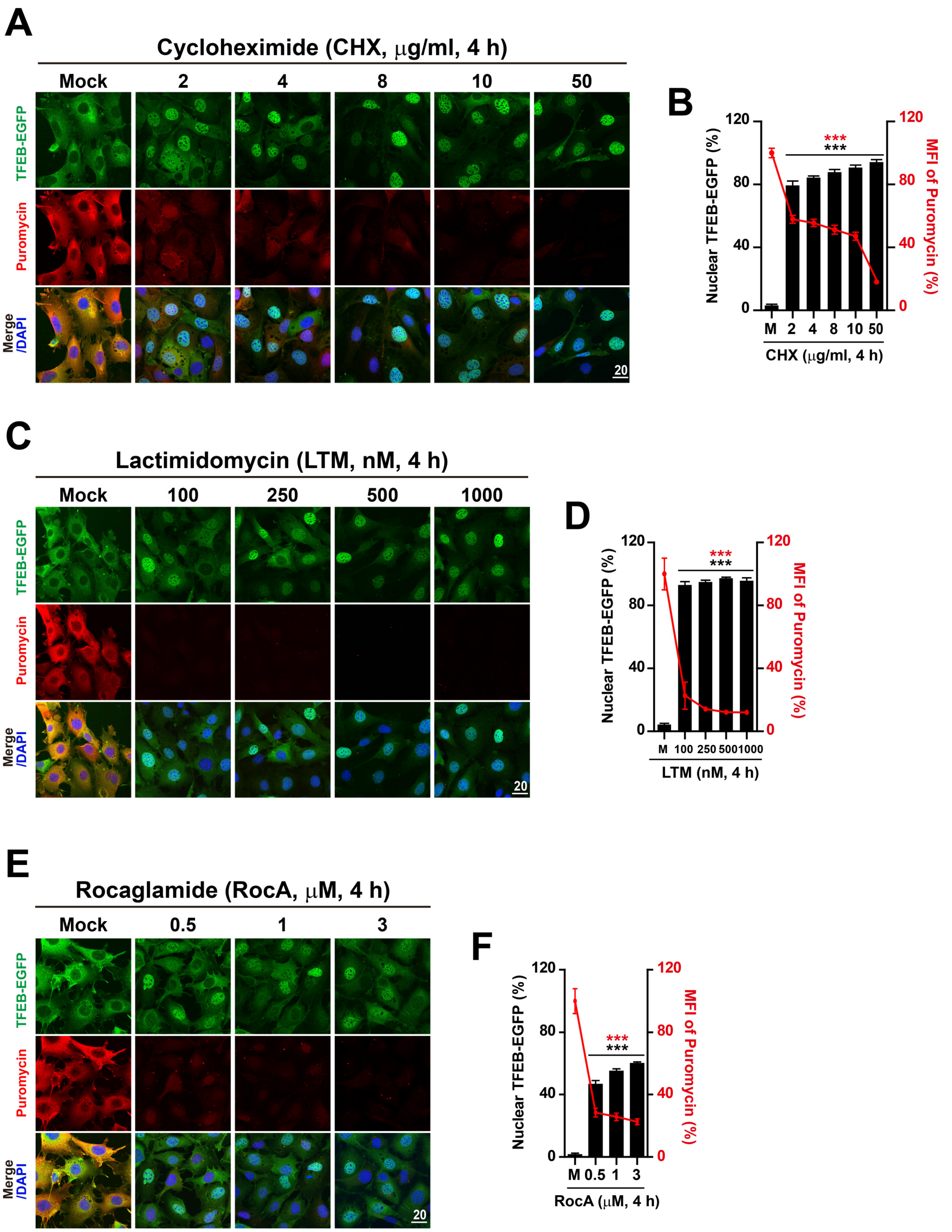

Figure S2. Translation inhibition induces TFEB nuclear translocation in a dose-dependent manner. **(A,C,E)** Representative images of confocal microscopic analysis. TFEB-EGFP-expressing MEF (MEF-TFEB-EGFP) cells were treated with CHX (A), LTM (C), or RocA (E) at the indicated concentrations for 4 h. Before harvesting, cells were additionally incubated with puromycin (10 µg/mL for 10 min) to label actively translating peptides. Cells were stained with anti-puromycin antibody (red) against puromycin-labeled proteins and DAPI (blue) for DNA. Cellular localization of TFEB-EGFP was indicated by green fluorescence in the cells. Scale bar, 20 µm. **(B,D,F)** Quantification of the percentage of MEF-TFEB-EGFP cells with nuclear TFEB-EGFP (left Y axis) and the mean fluorescent intensity (MFI) of puromycin (right Y axis) in (A), (C), or (E) images. Data are expressed as mean ± SEM of at least 150 cells from six random fields in each group. ***$p < 0.001$; 0 h vs. other concentrations.

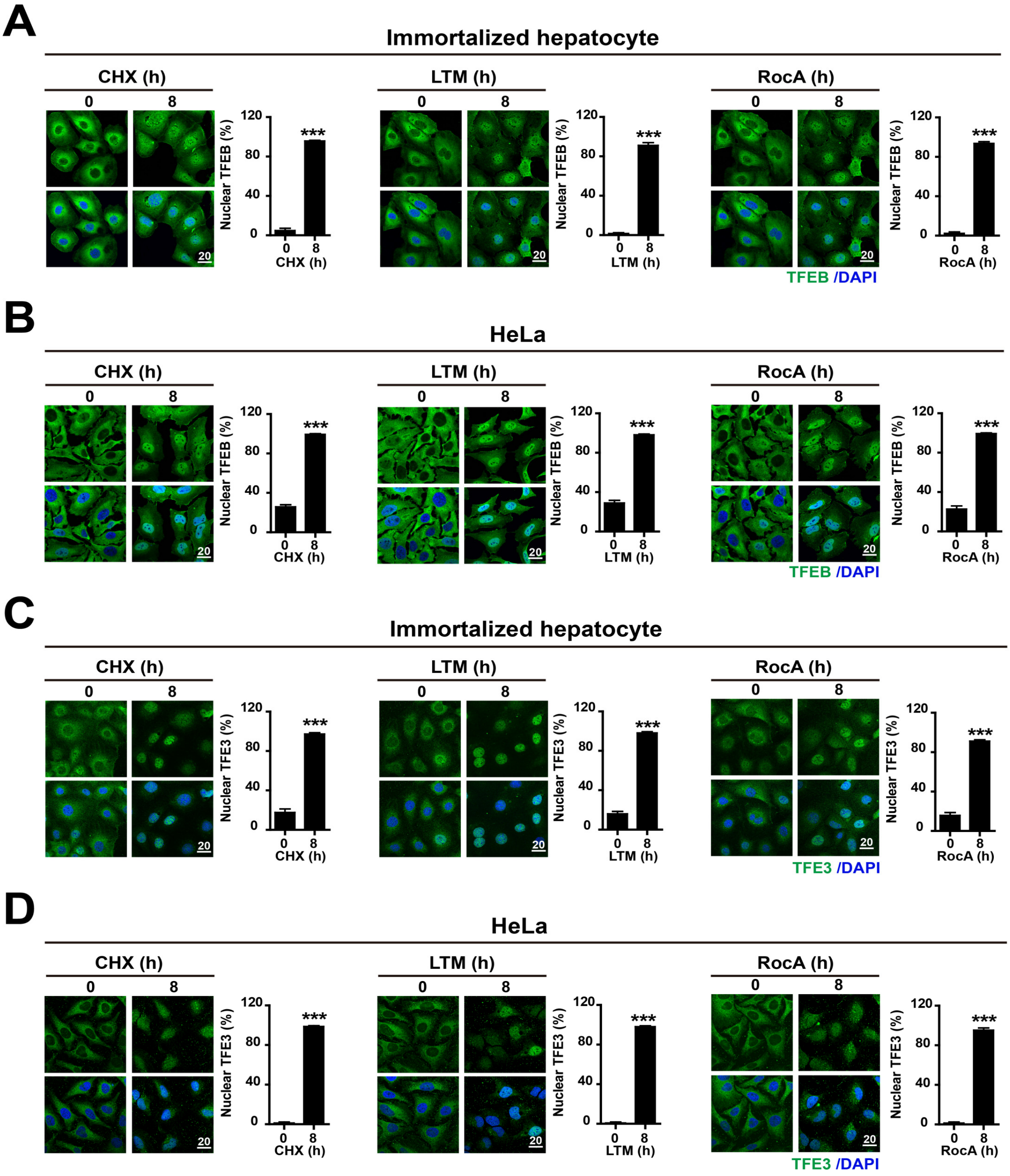

Figure S3. Translation inhibition induces TFEB and TFE3 nuclear translocation in immortalized hepatocytes or HeLa cells. **(A-D)** Representative images of the confocal analysis of TFEB (A,B) or TFE3 (C,D) subcellular distribution in different cell lines. Immortalized hepatocytes (A,C) or HeLa cells (B,D) were treated CHX (50 μg/mL), LTM (500 nM) or RocA (3 μM) for 0 and 8 h. Cells were fixed, permeabilized, and stained with anti-TFEB (A,B) (green) or anti-TFE3 (C,D) (green). DAPI (blue) indicated for nucleus in merged images (lower panels). Scale bar, 20 μm. The graphs represent quantification of the percentage of hepatocytes and HeLa with nuclear TFEB or TFE3 upon CHX, LTM or treatment. Data are expressed as mean ± SEM of at least 150 cells from six random fields in each group. ***p < 0.001; 0 h vs. 8 h.

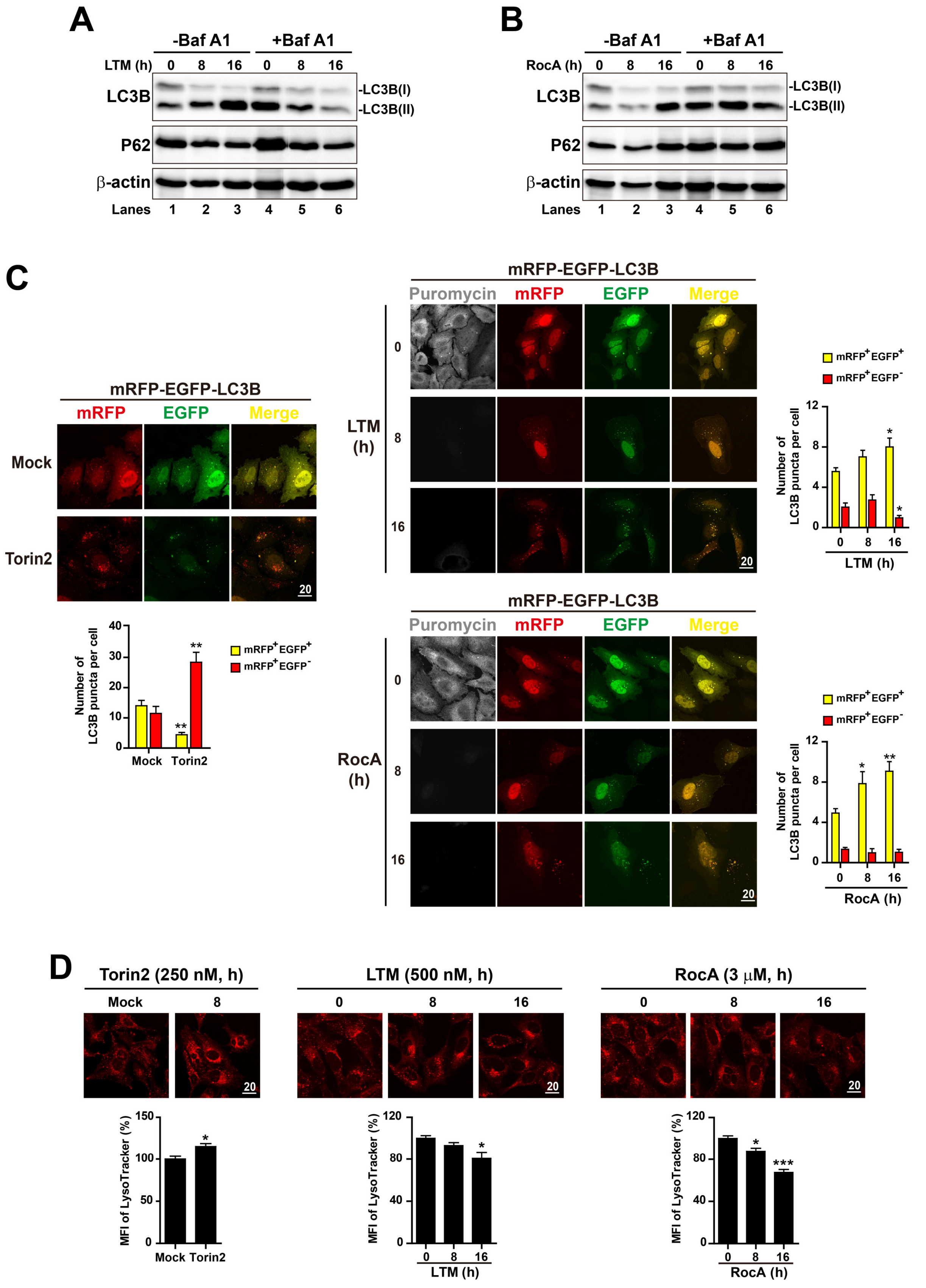

Figure S4. Translation inhibitors inhibit autophagic flux and induce lysosomal dysfunction. **(A,B)** Immunoblot analysis of protein lysates obtained from MEF-TFEB-EGFP cells treated with LTM (500 nM) (A) or RocA (3 μM) (B) for indicated times in

the absence or presence of lysosomal inhibitor bafilomycin A1 (Baf A1, 200 nM) for 3 h before harvest. The total cellular lysates were analyzed by immunoblotting with antibodies against LC3B, p62 or β-actin. **(C)** HeLa cells were transfected with a tandem fluorescent reporter pmRFP-EGFP-LC3B plasmid for 30 h and treated with LTM (500 nM) or RocA (3 μM) for indicated times and Torin2 (mTOR inhibitor, 250 nM) for 6 h as a positive control. Before harvesting, cells were additionally incubated with puromycin (10 μg/mL) for 10 min to label actively translating peptides. Cells were then stained with anti-puromycin antibody (gray) against puromycin-labeled proteins. Scale bar, 20 μm. Autophagic flux was analyzed graphically by quantifying the number of $mRFP^+EGFP^+$ (yellow LC3B puncta) as autophagosomes and $mRFP^+EGFP^-$ (red LC3B puncta) as autolysosomes. Data are expressed as mean ± SEM of at least 30 cells from six random fields in each group. *$p < 0.05$, **$p < 0.01$, and ***$p < 0.001$; 0 h vs. 8 h or 16 h. (**D)** HeLa cells were treated with LTM (500 nM) or RocA (3 μM) for indicated times and Torin2 (mTOR inhibitor, 250 nM) for 8 h as a positive control. Acidic vesicles were stained with LysoTracker Red (100 nM, 40 min) and the mean fluorescent intensity (MFI) was quantified and presented graphically. Scale bar, 20 μm. Data are expressed as mean ± SEM of at least 30 cells from six random fields in each group. *$p < 0.05$ and ***$p < 0.001$; 0 h vs. 8 h or 16 h.

Supplementary Table S1. The sequence of primers for qPCR.

Gene	Primer (Forward) sequence	Primer (Reverse) sequence
ATG5	CCAGGTGATGATTCACGG	GGCTGGGGGACAATGCTAA
ATG12	GGAGACACTCCTATAATGAAA	ATAAATAAACAACTGTTCCGA
LC3B	CGTCCTGGACAAGACCAAGT	ACCATCTACAGGAAGCCGTC
P62	GCTGCCCTATACCCACATCT	CGCCTTCATCCGAGAAAC
ATP6V1H	GGATGCTGCTGTCCCAACTAA	TCTCTTGCTTGTCCTCGGAAC
CTSB (cathepsin B)	ACAGTGCCACACAGCTTCTTC	TCCTTGATCCTTCTTTCTTGCC
CTSL (cathepsin L)	ATCAAACCTTTAGTGCAGAGTG	CTGTATTCCCCGTTGTGTAGC
HEXAB	CTGGTGTCGCTAGTGTCGC	CAGGGCCATGATGTCTCTTGT
LAMP1	ACCTGTCGAGTGGCAACTTCA	GGGCACAAGTGGTGGTGAG
LAMP2A	GCAGTGCAGATGAAGACAAC	AGTATGATGGCGCTTGAGAC
LAMP2B	GGTGCTGGTCTTTCAGGCTTGATT	ACCACCCAATCTAAGAGCAGGACT
LAMP2C	ATGTGCTGCTGACTCTGACCTCAA	TGGAAGCACGAGACTGGCTTGATT
MCOLN1	GCTGGGTTACTCTGATGGGTC	CCACCACGGACATAGGCATAC
TPP1	CCCCTCATGTGGATTTTGTGG	TGGTTCTGGACGTTGTCTTGG
UVRAG	CAAGCTGACAGAAAAGGAGCGAG	GGAAGAGTTTGCCTCAAGTCTGG
mTFEB	CCTGCCGACCTGACTCAGA	CTCAATTAGGTTGTGATTGTCTTTCTTC
hTFEB	ACCTGTCCGAGACCTATGGG	CGTCCAGACGCATAATGTTGTC
mTFE3	CCTGAAGGCATCTGTGGATT	TGTAGGTCCAGAAGGGCATC
β-ACTIN	GATCTGGCACCACACCTTCT	GGGGTGTTGAAGGTCTCAAA
